# A Case Report of Acute Heart Failure Due to Infective Aortic Endocarditis Diagnosed by Point-of-care Ultrasound

**DOI:** 10.5811/cpcem.2020.3.45002

**Published:** 2020-04-27

**Authors:** Ryan Gallagher, Michelle Wilson, Pamela Hite, Bradley Jackson

**Affiliations:** University of Kansas Health System, Department of Emergency Medicine, Kansas City, Kansas

**Keywords:** infective endocarditis, point-of-care-ultrasound, aortic valve vegetation, cardiac valve regurgitation

## Abstract

**Introduction:**

Infective endocarditis (IE) is a life-threatening condition with significant morbidity and mortality, and can require surgical repair.

**Case Report:**

A 36-year-old man presented to the emergency department for worsening dyspnea and chest pain. Point-of-care echocardiography demonstrated a mobile oscillating mass on the aortic valve with poor approximation of the valve leaflets, suggesting aortic valve insufficiency secondary to IE as the cause of acute heart failure. The patient underwent emergent aortic valve replacement within 24 hours.

**Discussion:**

While point-of-care echocardiography has been well documented in identifying tricuspid vegetations, aortic valve involvement and subsequent heart failure is less well described. Earlier recognition of aortic valve vegetations and insufficiency can expedite surgical intervention, with decreased complication rates linked to earlier antimicrobial therapy.

**Conclusion:**

This case report highlights the ability of point-of-care ultrasound to identify aortic vegetations, allowing for the earlier diagnosis and therapy.

## INTRODUCTION

Infectious endocarditis (IE) is a life-threatening condition that carries significant morbidity and mortality requiring prompt diagnosis, therapy, and sometimes-invasive interventions^1^ The proportion of IE patients undergoing surgery has increased over time to about 50%.^1^ Valve replacement rates due to IE have increased steadily from 2000–2007 from about 15 per 1000 cases to 25 per 1000 cases of IE, and then plateaued from 2007–2011.^2^ Between 2000 and 2011 there were 457,052 IE hospitalizations with a steady rise in incidence from 29,820 in 2000 to 47,134 in 2011.^2^ This rise is likely related to an increase in the prevalence of risk factors for IE including invasive procedures, intravenous drug use, human immunodeficiency virus, and diabetes.^1^ Also contributing to this rise is increased survival of predisposed populations such as those with congenital heart disease and prosthetic implants.^2^ Other contributing factors to the increased incidence of IE may be improvements in diagnostic methods as well as less-stringent recommendations for prophylactic antibiotic regimens by the American Heart Association in 2007.^3^,^4^

Duke criteria for the diagnosis of IE include pathologic specimens, typical organism growth on blood cultures, and evidence on echocardiography. As neither blood culture nor pathology results are available in the emergency department (ED), the diagnosis will always require evidence on point-of-care echocardiogram to be made. With patients in need of prompt antibiotic therapy and potentially emergent surgical intervention, this then raises the question of the capability of point-of-care ultrasound to detect IE.

## CASE REPORT

**Image 1:**
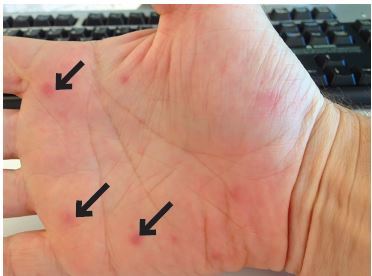
Palmar rash: Photograph of palmar lesions (arrows) provided by the patient consistent with Janeway lesions.

A 36-year-old man with no past medical history presented to the ED for worsening dyspnea, orthopnea, and chest pain in the context of three months of night sweats, unintentional weight loss, migratory arthralgia, myalgias, and recent palmar lesions (Image 1).

**Image 2:**
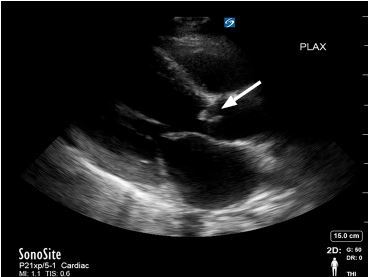
Aortic vegetation: Parasternal long-axis view showing a mobile oscillating mass on the aortic valve (arrow).

Outpatient rheumatologic and infectious workup had shown only an elevated erythrocyte sedimentation rate. Initial vital signs were notable for a blood pressure of 124/41 millimeters of mercury, heart rate 127 beats per minute, respiratory rate 20 breaths per minute, oxygen saturation of 98% on room air, and a temperature of 37.4 degrees Celsius. He appeared to be in a minimal amount of respiratory distress. Physical examination revealed a new pandiastolic murmur, lower lung field crackles, and bounding peripheral pulses. Electrocardiogram revealed sinus tachycardia without ST-segment abnormalities. Point-of-care echocardiography was performed, which noted a mobile oscillating mass on the aortic valve with poor approximation of the aortic valve leaflets on diastole (Image 2, Video).

The constellation of findings was suggestive of acute heart failure from aortic insufficiency due to likely IE of the aortic valve. IE was felt more likely because the finding met the Duke minor criteria of palmar lesions consistent with Janeway lesions, with the diagnosis fully confirmed when three blood cultures subsequently revealed *Streptococcus sanguinis*. The patient was admitted and underwent emergent aortic valve replacement less than 24 hours later with surgery describing the two coronary aortic valve cusps as obliterated by infection with greater than 1.5 millimeters mobile vegetation on the remaining non-coronary cusp.

## DISCUSSION

Point-of-care echocardiography provided key information, enabling the timely diagnosis of IE involving the aortic valve. While point-of-care echocardiography has previously been described as capable of identifying tricuspid vegetations,^5^,^6^ sensitivity in aortic vegetations is not well described. Further study would be needed to compare point-of-care transthoracic echocardiography (TTE) to cardiology TTE, which has been estimated to have a sensitivity for IE around 70% for native valves and 50% for prosthetic valves with a specificity of around 90%, according to the European Society of Cardiology in 2015.^7^ Similarly, a meta-analysis of 16 articles in 2017 on TTE found a sensitivity of 66% and a specificity of 95% for detecting IE on native valve^8^. These studies demonstrate the value of TTE in the workup for IE, despite not being as accurate as transesophageal echocardiography.^8^ Specific situations where TTE may not identify vegetations include underlying valvular thickening or calcification, prosthetic shadowing, recent vegetation migration or embolization, and poor acoustic windows secondary to obesity, hyperinflated lungs, or narrow interspaces.

**CPC-EM Capsule**

What do we already know about this clinical entity?

Infectious endocarditis is a life-threatening condition that carries significant morbidity and mortality, requiring sometimes-invasive interventions.

What makes this presentation of disease reportable?

While point-of-care echocardiography has been documented in identifying tricuspid vegetations, aortic valve involvement and subsequent heart failure is less well described.

What is the major learning point?

Point-of-care echocardiography is useful in undifferentiated heart failure, allowing for quicker diagnosis of underlying etiology and direction of therapy.

How might this improve emergency medicine practice?

Earlier recognition of aortic valve vegetations can expedite surgical intervention, with fewer complication rates linked to earlier antimicrobial therapy.

The potential for an emergency physician to achieve earlier recognition of cardiac valve vegetations is valuable because patients may require emergent surgical intervention, such as the valve replacement in this case, and as decreased complication rates have been linked to the earlier initiation of antimicrobial therapy. In a series of 1437 patients from the International Collaboration on Endocarditis in 2007, the embolic stroke rate decreased from 4.8 to 1.7 per 1000 patient years from the first to the second week of antibiotic treatment.^9^

Point-of-care echocardiography may also be useful for the detection of secondary valvular complications of endocarditis, obtaining prognostic indicators, and supporting the need for surgical intervention. The valvular complications that may be seen on echocardiography are regurgitation, valve perforation, and abscess or fistula formation due to destruction of tissue by bacterial invasion and proliferation.^1^ The main risk factor for complications is the length of the vegetation, with one retrospective cohort study finding the probability of sustaining a complication to be 10% when vegetations were 6 millimeters (mm) in size, 50% when lesions were 11 mm, and almost 100% when lesions were greater than or equal to 16 mm.^10^ Mortality has also been linked to vegetation length with one study of intravenous drug users with right-sided endocarditis demonstrating an increased mortality rate of 33% in patients with vegetations greater than 2 centimeters (cm) in length, compared to a mortality rate of only 1.3% in those with vegetations less than 2 cm in length.^11^

## CONCLUSION

Given the elusive nature of infective endocarditis, point-of-care echocardiography may not be used early enough in the diagnostic work-up. Point-of-care ultrasound was an invaluable bedside diagnostic tool in this patient with IE of the aortic valve. With a low index of suspicion to evaluate for vegetations and the knowledge that it is possible to find them on both the tricuspid and aortic valves, a critical diagnosis can be made in a timelier manner.

## Supplementary Information

Video Aortic vegetation: Parasternal long-axis and apical four-chamber view showing a mobile oscillating mass on the aortic valve.
